# Adaptation of the Golgi Apparatus in Cancer Cell Invasion and Metastasis

**DOI:** 10.3389/fcell.2021.806482

**Published:** 2021-12-10

**Authors:** Sarah Bui, Isabel Mejia, Begoña Díaz, Yanzhuang Wang

**Affiliations:** ^1^ Department of Molecular, Cellular and Developmental Biology, University of Michigan, Ann Arbor, MI, United States; ^2^ Department of Internal Medicine, Division of Medical Hematology and Oncology, The Lundquist Institute for Biomedical Innovation at Harbor-UCLA Medical Center, Torrance, CA, United States; ^3^ David Geffen School of Medicine and Jonsson Comprehensive Cancer Center, University of California, Los Angeles, Los Angeles, CA, United States; ^4^ Department of Neurology, University of Michigan School of Medicine, Ann Arbor, MI, United States

**Keywords:** Golgi, cancer, dissemination, metastasis, oncogenic transformation, proliferation, signaling, tumorigenesis

## Abstract

The Golgi apparatus plays a central role in normal cell physiology by promoting cell survival, facilitating proliferation, and enabling cell-cell communication and migration. These roles are partially mediated by well-known Golgi functions, including post-translational modifications, lipid biosynthesis, intracellular trafficking, and protein secretion. In addition, accumulating evidence indicates that the Golgi plays a critical role in sensing and integrating external and internal cues to promote cellular homeostasis. Indeed, the unique structure of the mammalian Golgi can be fine-tuned to adapt different Golgi functions to specific cellular needs. This is particularly relevant in the context of cancer, where unrestrained proliferation and aberrant survival and migration increase the demands in Golgi functions, as well as the need for Golgi-dependent sensing and adaptation to intrinsic and extrinsic stressors. Here, we review and discuss current understanding of how the structure and function of the Golgi apparatus is influenced by oncogenic transformation, and how this adaptation may facilitate cancer cell invasion and metastasis.

## 1 Introduction

The Golgi apparatus is an essential eukaryotic cell organelle that mediates protein and lipid glycosylation, lipid biosynthesis, intracellular trafficking, and protein secretion. In mammalian cells, the Golgi apparatus also functions as a microtubule organizing center, an intracellular signaling platform, and a stress sensor [for excellent recent reviews see (Mayinger, 2011; Sasaki and Yoshida, 2015; Wu and Akhmanova, 2017)]. All these functions are essential for cell proliferation, survival, migration, and cell-cell communication during normal physiology as well as during cancer progression. In cancer cells, the functions of membrane organelles, including the endoplasmic reticulum (ER), mitochondria and lysosomes, are adapted to support continuous proliferation and evasion from cell death. However, the adaptation of the Golgi apparatus in cancer cells has been less extensively studied.

The morphology of the Golgi apparatus is dynamic, and becomes profoundly altered during cell division, when the stacked Golgi structure completely disassembles and reforms in the daughter cells. During interphase, its complex architecture is linked to its function. For instance, glycosylation and cargo sorting are defective when Golgi is experimentally forced to disassemble in interphase (Xiang et al., 2013; Bekier et al., 2017). Furthermore, the Golgi in mammalian cells forms a ribbon in which several cisternae stacks link laterally. The Golgi ribbon appears to be important for the non-classical functions of the mammalian Golgi on intracellular signaling and stress sensing (Makhoul et al., 2018).

Here, we review and discuss our current knowledge on the morphological and functional adaptations of the Golgi apparatus during cancer invasion and metastasis. First, we will briefly introduce the different Golgi morphologies of cancer cells. In the following sections, we emphasize the role of the Golgi in each step of the metastatic cascade by presenting the most representative studies that convey its multifaceted participation in cancer progression. We highlight examples of Golgi-specific proteins regulating metastatic phenotypes and discuss how these conceptual advances in understanding Golgi in cancer can guide us in developing better diagnostic tools and therapeutic options in the future.

## 2 The Golgi Morphology in Cancer Cells

**FIGURE 1:**
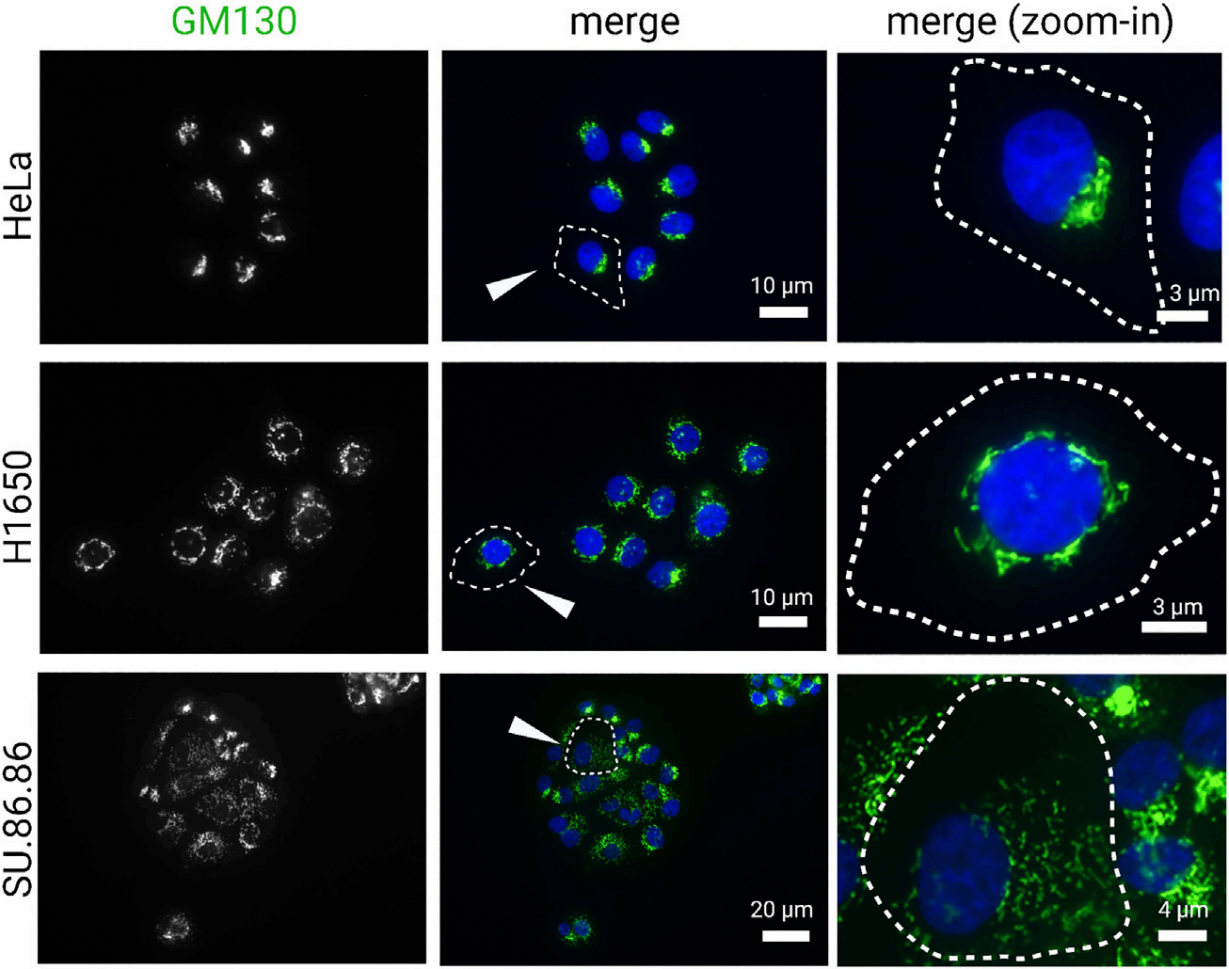
Golgi morphology in cancer cell lines. Carcinoma cell lines, HeLa (cervical), H1650 (lung), and SU.86.86 (pancreatic), were stained for GM130 (cis-Golgi marker) and DAPI (blue). Images represent distinct Golgi morphologies in different carcinoma cell lines. Arrowheads indicate outlined cells that are magnified in the right column (zoom-in).

The exact feature of Golgi in cancer cells remains debated; although it is clear that both the structure and function of this organelle is affected by the hallmarks of cancer, which in turn can also promote those fundamental traits of cancer by causing aberrant glycosylation, enhancing survival and proliferation, and increasing migration. Early studies described abnormal morphology as a common feature of the Golgi in cancer (Maldonado et al., 1966; Kellokumpu et al., 2002; Petrosyan, 2015). The Golgi in cancer cells often appears as constitutively disassembled in discrete units distributed throughout the cytoplasm. In some cancer cells, cisternal stacks are maintained, but they lack its characteristic ribbon organization (Figure 1). In other cases, the stacks themselves are disassembled, or even fragmented (Figure 1). Lastly, the Golgi morphology in some cancer cells is indistinguishable from non-cancerous cells (Figure 1). It remains unclear to what extent the alteration in Golgi morphology of cancer cells reflects a functional adaptation that actively contributes to malignancy or simply a bystander effect of cancer cell transformation. Recent studies suggest that dynamic regulation of Golgi morphology may indeed contribute to cancer malignancy.

### 2.1 Golgi Fragmentation in Cancer

Given the wide range of Golgi morphologies observed across cancer types, we will first briefly summarize the molecular changes regulating Golgi structure that generally coincide with cancer progression.

*Golgi dispersal in cancer cells*—Cancer cell lines across a wide variety of cancer types, including colon (Egea et al., 1993; Kellokumpu et al., 2002), breast (Sewell et al., 2006), gastric (He et al., 2010), and prostate cancer cells (Petrosyan et al., 2014; Nolfi et al., 2020), display fragmented Golgi. In tissue culture, the Golgi ribbon is intact in many cancer cell lines, but it undergoes morphological change upon the induction of epithelial-to-mesenchymal transition (EMT) or migration (Bisel et al., 2008b). Golgi fragmentation is often seen in various tumor tissue types from patients (Maldonado et al., 1966; Tani et al., 1975; Castejón, 1999; Kellokumpu et al., 2002; Sano et al., 2002; Zhang, 2021). Frozen tissue sections showed intact Golgi structure in normal colon epithelial cells, in contrast to small punctate Golgi structure found in cancerous colon epithelium (Kellokumpu et al., 2002). In pituitary tumors, Golgi has a distinct ultrastructural appearance described as “honeycomb” (Sano et al., 2002). Fenestration of Golgi cisternae were reported in human brain tumors (Tani et al., 1975) and hypertrophy of the Golgi in melanoma cells (Maldonado et al., 1966). It is generally agreed that an organized Golgi structure is required for accurate protein glycosylation and sorting, and our study revealed that Golgi fragmentation accelerates protein trafficking and cell proliferation (Xiang et al., 2013; Zhang and Wang, 2016; Bekier et al., 2017). Consistently, alteration of Golgi functions such as glycosylation is often seen in cancer cells, as discussed below. Taken together, Golgi disorganization, ranging from mild enlargement to vesicular dispersal, has been linked to the orchestration of molecular pathways related to cell survival, cell cycle progression, and cell migration – all major hallmarks of cancer.

*Golgi structure formation and regulation*—The basic unit of the Golgi is comprised of stacked cisternae that are highly polarized and aligned to receive cargo from the ER and sort cargo for Golgi export. Golgi structural formation is dependent on a network of Golgi structural proteins, also known as Golgi matrix proteins, that function to hold adjacent cisternae together. Two important Golgi matrix proteins: GRASP65 and GRASP55 (Golgi reassembly stacking protein of 65 kDa/55 kDa) were originally identified as Golgi stacking factors (Barr et al., 1997; Shorter et al., 1999). GRASP65 and GRASP55, found mainly at the *cis* side of the Golgi stack and *medial-trans* region, respectively, homodimerize and further *trans*-oligomerize to facilitate Golgi stacking and ribbon linking (Wang et al., 2003; Tang et al., 2016). Phosphorylation of GRASP55 and GRASP65 on a number of serine and threonine residues at its C-terminus results in unlinking of the Golgi ribbon and dissociating the cisternae in the stacks (Tang et al., 2012). It has been shown that GRASP65 is the major target of cdc2 (in complex with cyclin B) and polo-like kinase (plk) (Lin et al., 2000; Wang et al., 2003) - two major mitotic kinases. GRASP65 phosphorylation leads to Golgi cisternal unstacking (Wang et al., 2003), while GRASP65 dephosphorylation by the protein phosphatase PP2A allows the reformation of the Golgi stack (Tang et al., 2008).

*Golgi dispersal and kinase activation in cancer cells*—Mitotic phosphorylation of Golgi structural proteins results in Golgi disassembly in mitosis. Likewise, kinase activation by proinflammatory cytokines, cellular stresses, and growth factors can also trigger Golgi fragmentation. For example, PKCα has recently been identified as a kinase that directly phosphorylates GRASP55 during thapsigargin or histamine treatment (Ireland S. et al., 2020). Whether GRASPs can be phosphorylated by other kinases implicated in cancer remains to be studied, however, a correlative link between aberrant kinase activation and disruption of Golgi morphology has been noted (examples below). Reduced levels of a diacylglycerol kinase, DGKZ, in p53-mutant breast cancer cells subsequently accumulated diacylglycerol at the Golgi membranes and induced Golgi tubulo-vesiculation through local PKD activation (Ching et al., 2007; Weller et al., 2010; Capaci et al., 2020). Reports of fragmented Golgi in ovarian and breast cancer were correlated with Rab25 overexpression and kinase dysregulation (Cheng et al., 2004; Davidson et al., 2006; Yin et al., 2012). In other tumor cells, Src, ERK8, and p21-activated protein kinase (Pak1) were shown to have elevated expression (Ching et al., 2007; Chia et al., 2014). Activation of MAP kinase (mitogen activated protein kinase)/ERK2 (the extracellular-signal-regulated kinase) also causes Golgi fragmentation (Tang et al., 2012; Kienzle et al., 2013; Ireland et al., 2020). Post-mitotic Golgi reassembly is mediated by membrane fusion via p97 and NSF, with their adaptor proteins. In Wilms’ tumor and other types of cancers, HACE1, a ubiquitin ligase that regulates p97-mediated Golgi membrane fusion following mitosis (Tang et al., 2011), has been identified as a tumor suppressor that is downregulated, which may further contribute to the fragmented Golgi phenotype (Anglesio et al., 2004; El-Naggar et al., 2019). Given that kinases are involved in diverse signaling pathways, including mitogenic signaling, it will be important to de-couple cancer cell changes in cell cycle progression and mitotic Golgi disassembly to better understand how the Golgi structure is affected in cancer pathogenesis.

### 2.2 Other Golgi Phenotypes in Cancer

Overexpression of Golgi matrix proteins such as GRASP55 and GM130 have been correlated with poor prognosis in some cancer patients, suggesting that they have important functions in tumor cells. However, it is unclear if the Golgi in these tumors with high expression of Golgi matrix proteins display compact Golgi phenotypes. GRASP55 was shown to be hypomethylated and hence overexpressed in invasive adenocarcinoma (Husni et al., 2019). GM130 expression is positively correlated with invasion and poor prognosis in lung and gastric tumors (Chang et al., 2012; Zhao et al., 2015). GOLIM4 was shown to promote tumorigenesis in nasopharyngeal carcinoma through maintaining the organization of Golgi (Luo et al., 2021). Golgi compaction in cancers has been linked to EMT (Tan et al., 2017). Cancer EMT is considered now as a spectrum of hybrid or partial E/M states, rather than a two-state paradigm; therefore, it is reasonable to hypothesize that if Golgi morphology is correlated with epithelial and mesenchymal states, then Golgi architecture would span a range of structures in varying degrees of dispersal as well as compaction. Without complete fragmentation, the Golgi can be fenestrated (Tani et al., 1975), “honey-comb” patterned (Sano et al., 2002), or extended phenotype (Halberg et al., 2016) in different tumors. It is becoming more apparent, as more studies are investigating the role of Golgi in cancer, that this dynamic organelle is at the center of the many biological processes involved in metastasis.

## 3 The Molecular Mechanism of Golgi Adaptation in Metastasis

### 3.1 The Metastatic Cascade

**FIGURE 2:**
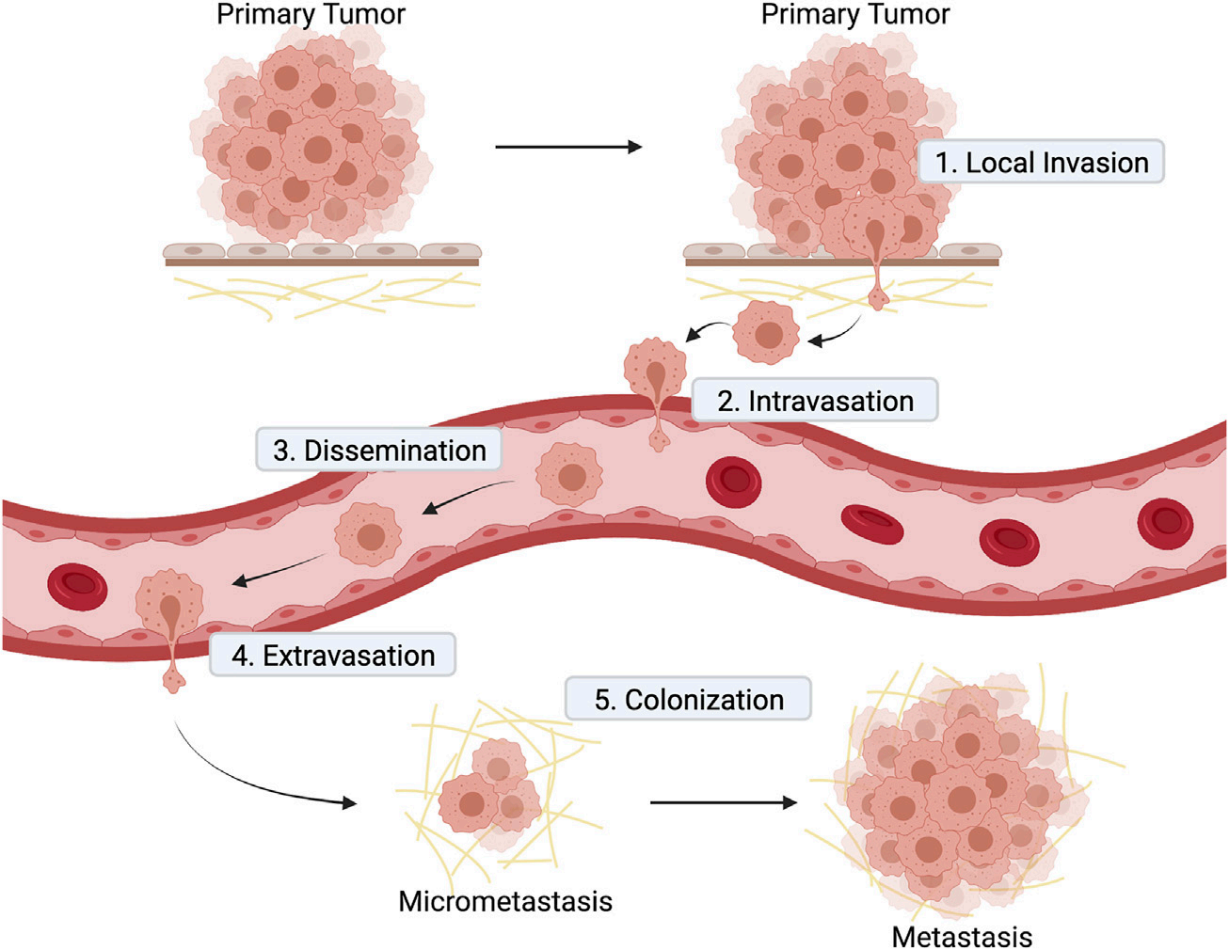
Diagram illustrating the main steps of metastatic cascade. 1) Local invasion: cells at the primary tumor site invade the surrounding stroma; 2) intravasation of cancer cell into blood vessels; 3) dissemination of cancer cells in blood vessels to distant sites; 4) extravasation of cancer cell from blood vessel to a secondary tissue; 5) colonization of a secondary tissue to form metastasis.

Metastasis is an extremely complex pathological process and understanding it is fundamental to achieve the elusive goal of finding curative treatments for advanced cancers. The metastatic cascade model (recently reviewed in (Lambert et al., 2017) presents metastasis as a stepwise process (Figure 2) that starts when a subset of cancer cells residing in the primary tumor acquire invasive capacity. Acquisition of aberrant invasiveness enables cancer cells from solid tumors to travel through the surrounding tissues and reach the blood or lymphatic circulatory systems for metastatic dissemination. After entering the vessels by a process called intravasation, those cancer cells that survive in circulation and successfully leave the circulation (extravasation) at distant sites may subsequently form micrometastasis (metastatic colonization). When disseminated cancer cells proliferate to form macroscopic secondary tumors, the metastatic cascade is completed (Figure 2).

At each step of the metastatic cascade, cancer cell intrinsic factors, such as driver mutations in oncogenes and/or tumor suppressors, cooperate with cancer cell extrinsic factors, such as signals from the tumor microenvironment, to promote metastasis (Lambert et al., 2017). The tumor microenvironment is comprised of cellular and non-cellular components, and the crosstalk between these and the cancer cells not only promotes the growth of the primary tumor, but also its metastatic competency. The reciprocal interactions between cancer cells and the tumor microenvironment may be established through communication by either direct contact (cell-matrix or cell-cell), or by indirect contact (autocrine, juxtacrine and paracrine signaling). Activation of cell surface receptors by ligands is central to the above communication mechanisms and is often coupled with aberrant remodeling of the extracellular matrix during invasion and metastasis. Cell surface receptors, secreted and transmembrane ligands, matrix proteases and extracellular vesicles are all essential for the cross-talk between cancer cells and the tumor microenvironment, and, therefore, modulate one or more steps of the metastatic cascade.

Most of these molecules are processed and sorted in the Golgi apparatus, placing this organelle at a central position in the regulation of invasion and metastasis. Importantly, recent findings indicate that the central role of the Golgi apparatus in sustaining invasion and metastasis goes beyond its function in receptor and ligand post-translational modifications, lipid biosynthesis, intracellular trafficking, protein secretion, and formation of extracellular vesicles (Petrosyan, 2015). As mentioned earlier, the Golgi apparatus is also an intracellular signaling platform (Chiu et al., 2002) and a homeostasis sensor that detects and responds to stress (Zhang et al., 2018; Zhang et al., 2019; Ireland S. C. et al., 2020). These non-classical Golgi functions are currently the focus of active investigation during disease states and appear highly relevant during cancer invasion and metastasis.

### 3.2 Golgi Adaptation During Cancer Cell Invasion

#### 3.2.1 Epithelial to Mesenchymal Transition

**FIGURE 3:**
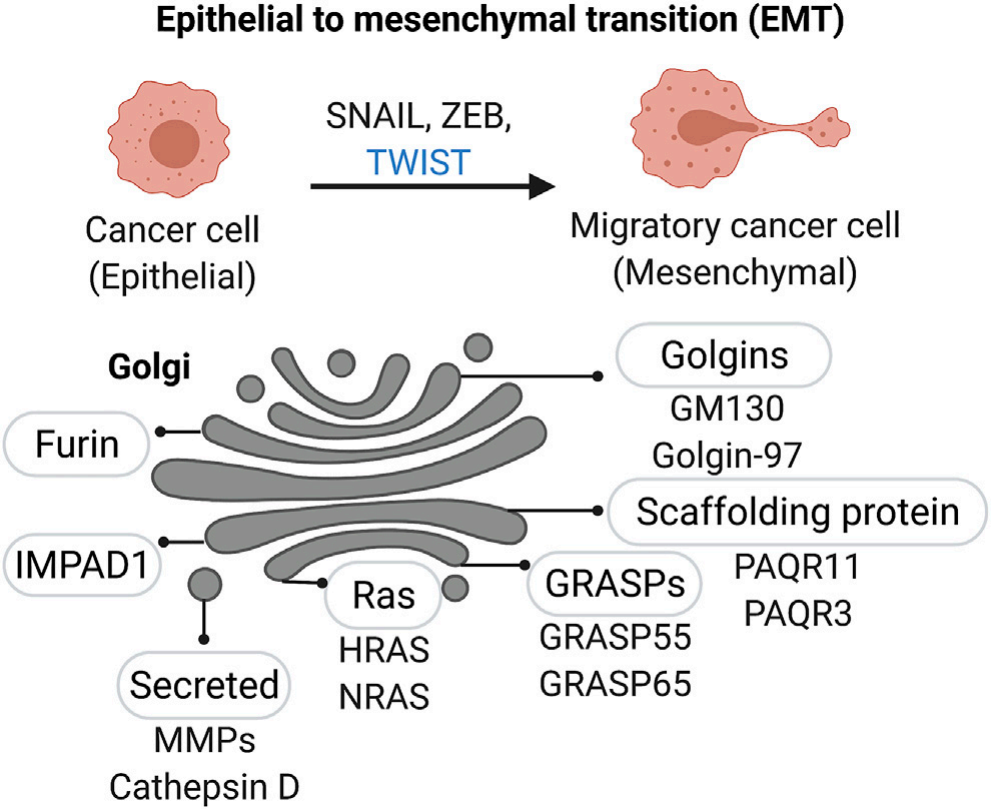
Golgi associated factors in Epithelial-to-Mesenchymal Transition (EMT). A number of Golgi-localized proteins, including Golgi matrix proteins and enzymes, are linked to changes in Golgi structure and function as cancer cells acquire a mesenchymal-like migratory phenotype.

*EMT and transcriptional regulation -* Cell invasion into the tissues surrounding the primary cancer lesion is the first step of the metastatic cascade. In the case of solid tumors of epithelial origin, which represent the majority of human cancers, the acquisition of invasive capacity is associated with aberrant reactivation of the developmental process of epithelial to mesenchymal transition (EMT), recently reviewed in (Dongre and Weinberg, 2019). This process is characterized by the loss of cell-cell contact and the acquisition of cytoskeletal changes that facilitate cell motility (Figure 3). Expression of a set of specific transcription factors, including SNAIL, TWIST, and ZEB families, drives the EMT program during development and cancer progression. Interestingly, PAQR3 a Golgi scaffolding protein (also named RKTG for Raf kinase trapping to the Golgi) is actively involved in the degradation of Twist1, a critical transcription factor required for the initiation of EMT and metastasis of tumor cells (Guo et al., 2016). Although well-studied instances of Golgi directly regulating EMT-associated transcription factors are not available, it is known that EMT during cancer progression is aberrantly activated by a variety of factors, including amino acid starvation—to which the Golgi is highly responsive. Once the invading cells reach their final destination, they often partially reverse their EMT phenotype. However, as mentioned earlier, EMT in cancer is not completely resolved and cancer cells often retain a mixture of mesenchymal and epithelial traits which confer these cells with the necessary plasticity to adapt to changing environments (Yang et al., 2020).

*Pro-tumorigenic secretion during EMT*—Interestingly, compaction of the Golgi apparatus has recently been discovered as part of the EMT program (Tan et al., 2017). Enhanced Golgi ribbon linking and stack formation through Golgi scaffolding protein PAQR11 (progestin and adipoQ receptor 11) enables Golgi compaction. Furthermore, p53 loss up-regulates the expression of PAQR11 and promotes a pro-tumorigenic secretory phenotype by recruiting ARF1 to Golgi membranes, which facilitates cargo-loading into vesicles destined for the extracellular space. In particular, the secretion of PLAU, a protease that prevents the induction of apoptosis in cells upon loss of attachment to the extracellular matrix (ECM) and neighboring cells during EMT, is dependent on PAQR11 (Tan et al., 2021).

*Alteration of Golgi glycosylation enzymes during cancer invasion*—During cancer progression, EMT contributes to malignancy, as dissolution of cell-cell junctions allow cancer cells to detach from the primary tumor, migrate, and survive in transit to secondary sites of metastasis. A typical example of cancer-related EMT is cadherin switching between E- and N-cadherins. Interestingly, E-cadherin trafficking to the plasma membrane is dependent on Golgin-97 (Lock et al., 2005), a tethering factor in the trans-Golgi network (TGN). Increased N-glycosylation of E-cadherin with bisecting GlcNAc structures by Golgi resident enzyme Gn-TIII (N-acetylglucosaminyltransferase III) promotes E-cadherin cell surface stability and function (Yoshimura et al., 1996; Pinho et al., 2012). During EMT, expression of GnT-III and its bisecting GlcNAc structures is reduced; while GnT-V and its 1,6 GlcNAc branching structures, which have been associated with enhanced malignancy and metastasis, are increased (Asada et al., 1997; Granovsky et al., 2000; Gu and Taniguchi, 2008; Gu et al., 2009). Remarkably, a recent study showed that methylation and demethylation of the promoter are involved in regulating the expression of GnT-III during EMT and MET, respectively. Knockdown of GalNAc-T3 (GALNT3) can initiate EMT through aberrant E-cadherin glycosylation (Raghu et al., 2019).

*RAS activation at the Golgi during EMT -* Other oncogenes such as the Ras family, encoded by the *KRAS, HRAS,* and *NRAS* genes, are also integral molecular players in cancer cell invasion. Recent evidence supports the function of Ras oncoproteins in tumor cell acquisition of EMT features. Ras signaling through phosphoinositide 3-kinase (PI3-Kinase) and Raf Kinase Inhibitory Protein (RKIP) cooperatively regulate epithelial cell plasticity and fine tunes cancer cell motility (Schuierer et al., 2004; Tripathi and Garg, 2018). The Ras family are among the most frequently altered and some of the earliest described oncogenes in human malignancies. They play numerous functions not limited to supporting proliferative signaling, survival pathways, metabolic and immunologic functions; as well as regulating cell morphology, differentiation, cellular adhesion, cell invasion and migration. Ras proteins have been found on the plasma membrane, in the endoplasmic reticulum, endosomal network, mitochondria, and the Golgi apparatus. Interestingly, the level of each protein in these subcellular compartments varies according to their total abundance and between cell types. For example, it was reported that N-Ras and H-Ras maintain the highest Golgi pool (Chiu et al., 2002) (Figure 3). H- and N-Ras traffic between the Golgi and the plasma membrane (PM) depending on their acylation status (Misaki et al., 2010). H-Ras has two palmitoylation sites (Cys residues 181 and 184) and is distributed throughout the Golgi stacks. N-Ras contains one palmitoylation site (C181) and is found mainly at the *trans*-Golgi (Lynch et al., 2015). H/N-Ras get palmitoylated at the Golgi apparatus by DHHC9 and then transferred to the PM via the secretory pathway (Swarthout et al., 2005). After de-palmitoylation, H/N-Ras traffic back to the Golgi to be re-acylated. Selectively activated RAS at the Golgi by apoptogenic stimuli elicits cell death and prevents cellular transformation (Casar et al., 2018). Treatment with Palmostatin B reportedly blocks the Ras S-palmitoylation cycle, restoring epithelial-like features and induces dose-dependent cell death in N-Ras mutant melanoma cells (Vujic et al., 2016). Taken together, the Golgi can be regarded as a signaling platform from which proteins like Ras can be activated during EMT.

*Golgi positioning and directional cell migration*—With relevance to cancer invasion, cell polarity and directional cell migration are highly associated with Golgi positioning and structure. For example, the Golgi ribbon is unlinked and relocated to the side of the nucleus that faces the leading-edge during fibroblast directed cell migration (Pouthas et al., 2008). It is thought that this positioning facilitates polarized transport of proteins such as integrins and lipids, to the growing lamellipodium (Bisel et al., 2008a). In a different study, increased cell migration was associated with a stable placement of the Golgi apparatus rather than its position at the rear or front of the nucleus (Natividad et al., 2018). And alternatively, when cells are forced to migrate along a linear fibronectin pattern, the Golgi is positioned behind the nucleus due to geometrical constraints (Pouthas et al., 2008). Hence, one explanation for the inconclusive position of the Golgi during cell migration may stem from the leading edge spreading further in a typical wounding assay when the substrate is scratched, allowing for the Golgi to preferentially face the leading edge (Millarte and Farhan, 2012). Therefore, it seems that future studies on Golgi positioning during cell migration using traditional methods of scratch assays on 2D substrates, as well as in 3D cell culture models, will provide clarity on this outstanding question.

Repositioning of the Golgi and dynamic alterations of the organelle is carried out by a number of factors including the actin/microtubule network and their associated molecular motors in conjunction with Golgi proteins. Several Golgi proteins are known to regulate cell migration and polarization, including GM130 and GRASPs (Bisel et al., 2008a) (Figure 3). Phosphorylation of GRASP65 results in Golgi unstacking and reorganization at the leading edge of the migrating cell. GRASP65 mutants lacking the phosphorylation site prevented Golgi and centrosome reorientation during cell migration. Similarly, GM130 recruits YSK1 and activates the kinase which phosphorylates downstream cell polarity targets (Preisinger et al., 2004). Inactive YSK1 abrogates Golgi and centrosome reorientation during cell migration. GM130 also interacts with the Rho family GTPase Cdc42, a key regulator of cell polarization (Etienne-Manneville, 2004; Kodani et al., 2008; Baschieri et al., 2014). Loss of GM130 decreased angiogenesis and cancer cell invasion *in vitro* and suppressed tumorigenesis in a lung cancer mouse model (Chang et al., 2012). Moreover, GM130 expression positively correlated with pathological EMT and invasion of gastric cancer cells through Snail signaling (Zhao et al., 2015). However, in a breast and colorectal cancer model, when the authors silenced GM130, it was sufficient to induce E-cadherin downregulation (Baschieri et al., 2014). Moreover, it was demonstrated that depletion of GM130 increased cellular velocity and the invasiveness of breast cancer cells. Conversely, knockdown of cis-Golgi proteins golgin-160 and GMAP210 was reported fragment the Golgi into several mini-stacks and inhibit cell migration (Yadav et al., 2009). Therefore, it seems that the effect of different golgins may be cell-type dependent and perhaps even migration-type dependent (single cell migration as compared to collective cell migration). Although we continue to discover the contributions of different golgins to the cancer cell phenotype, these studies underscore the important roles of golgins in modulating tumorigenesis during EMT and the early steps of the metastatic cascade (Figure 3).

#### 3.2.2 Extracellular Matrix Remodeling

The ECM is comprised of 43 collagen subunits, 36 proteoglycans and ∼200 complex glycoproteins – altogether, amounting to roughly 300 macromolecules which constitute the core matrisome (Hynes and Naba, 2012). The extracellular matrix is a dynamic scaffold for cells and serves as mechanical support to maintain tissue integrity and elasticity. But function of the ECM goes beyond this physical platform for cells to reside; due to cell-matrix interactions, signaling cues from cell-surface receptor binding of ECM components modulate key cancer-associated cellular behavior such as proliferation, differentiation, and migration. The Golgi aids in the production of extracellular matrix (ECM) components and activation of key ECM remodeling proteins as tumor cells invade through the basement membrane and migrate through the tumor stroma.

*Golgi and enhanced ECM secretion during tumorigenesis*—The capacity of the cell to accommodate the enhanced secretion of ECM proteins during tumorigenesis involves a coordinated increase in secretory vesicle biogenesis. Interestingly, Kurie and colleagues recently demonstrated that GRASP55 is part of a Golgin-45/myosinIIA-containing protein complex which activates secretory vesicle biogenesis in the Golgi. In addition, loss of the tumor suppressor gene, p53, up-regulates the expression of the *trans*-Golgi network localized protein PAQR11, which recruits an adenosine diphosphate ribosylation factor 1–containing protein complex that loads cargos into secretory vesicles (Tan et al., 2021) (Figure 3). Accordingly, depletion of PAQR11 in human lung adenocarcinoma cells reduced the secretion of proteins - such as the collagen-modifying enzyme procollagen-lysine,2-oxoglutarate 5-dioxygenase 3 (PLOD3) – that were enriched in Gene Ontology terms, “ECM degradation,” and “proteolysis regulation”.

*ECM remodeling during tumor invasion*—In addition to the core matrisome, proteases and other enzymes are also secreted into the extracellular space and participate in the constant remodeling of the ECM to maintain homeostasis of the interstitial matrix and basement membrane. The cycle of ECM remodeling is the balance between deposition of ECM components, and its regulated degradation. Dysregulated ECM composition, stiffness, abundance, and organization contributes to many pathological conditions including cancer. Some of the well-known ECM regulating proteins include collagen crosslinking proteins - like nidogen, lysyl oxidase, and lysyl-hydroxylases, increase matrix stiffness. Conversely, other secreted proteins, such as metalloproteinases and cathepsins, degrade ECM. The main enzymes responsible for ECM degradation are matrix metalloproteinases, such as ADAMs and memprins, which belong to the metzincin enzyme family. There are 23 identified human MMPs that collectively can degrade all ECM proteins [see detailed review (Bonnans et al., 2014)]. Often observed upregulated in primary tumors and/or metastases are MMP-1, -2, -3, -7, -9, -13, and -14 (Egeblad and Werb, 2002; Deryugina and Quigley, 2015; Winer et al., 2018). Some other MMPs are also implicated in poor prognoses associated with human cancers.

*The role of the Golgi in ECM remodeling during tumor invasion*—Interestingly, MMPs1, 2, and 9 secretion was enhanced when a Golgi-localized protein, IMPAD1, was overexpressed in lung adenocarcinoma cells (Bajaj et al., 2020). The authors concluded that IMPAD1 modulates the Golgi-mediated secretion of proteases such as MMPs into the ECM, to promote lung cancer migration and invasion. In addition to secretion, the Golgi also controls two important post-translational modifications (PTMs) of MMPs: glycosylation and proteolytic cleavage. The MMPs exhibit high protein structure homology that can be separated into three domains: the pro-peptide, the catalytic domain, and the haemopexin-like C-terminal domain linked to the catalytic domain by a flexible hinge region. Each domain effectuates a specific activity for the protein and can be modified by highly specific PTMs. Post-translational modifications significantly expand the functionality and fine-tune both enzyme and substrate protein structure, subcellular localization, protein–protein interactions, and degradation [recently reviewed in (Madzharova et al., 2019)].

*MMP glycosylation in the Golgi*—All MMPs have potential glycosylation sites, with a handful of sites validated with known functions. MMP-9 is a well-characterized glycosylated MMP and is the most extensively glycosylated of all the MMPs (Madzharova et al., 2019). The protease contains 2*N-*linked glycosylation sites: Asn38 in the pro-domain and Asn120 at the catalytic domain. In addition, the region between the active site and the hemopexin domain is denoted as the OG domain because it contains 14 potential O-Glycosylation sites. The Golgi targeting mechanisms of core 1 β3 galactosyltransferase (C1GalT-1) and core 2N-acetylglucosaminyltransferase 2/M (C2GnT-2/M), which participate in the early O-glycosylation steps, are carried in vesicular complexes that utilize different golgins for docking to the Golgi: C2GnT-M-VC uses Giantin, while C1GalT1-VC employs the GM130-GRASP65 complex (Petrosyan et al., 2012). Notably, when GalNAc-T1 (GALNT1) is mislocalized from the Golgi to ER, consequential increase in O-glycosylation activates the matrix metalloproteinase MMP14 to promote tumor spread (Nguyen et al., 2017). Based on predictive models and comparison of conserved glycosylation sites near the active site across the MMP family, it is hypothesized that glycosylation of MMPs may serve as an additional layer to diversify the activity of these similar zymogens.

*MMP processing in the Golgi*—Although most MMPs are secreted into the extracellular space, six members of the MMP family are membrane anchored, also known as membrane-type MMPs (MT-MMPs). MMPs can be grouped into four major groups: gelatinases, matrilysins, archetypal MMPs, and furin-activatable MMPs. All six membrane-type MMPs (MMP-14, -15, -16, -17, -24, and -25) have a furin cleavage site in the pro-peptide, which is a feature also shared by MMP-11 (Pei and Weiss, 1995; Ra and Parks, 2007). Furin is enriched at the Golgi (Figure 3) and cleaves proteins just downstream of a basic amino acid target sequence [canonically, Arg-X-(Arg/Lys) -Arg'] (Sato et al., 1996). Interestingly, MMP-14 activation by furin has been shown to depend on GRASP55. Overexpression of GRASP55 increased MMP-14 and furin complex formation; the study suggests that GRASP55 serves as an adaptor protein coupling MMP-14 with furin since it was shown to interact directly with MMP-14 at the LLY^573^ motif (Roghi et al., 2010). Hence, not only is Golgi home to important proteases that participate in activation of MMPs, but that GRASPs and other golgins, can moonlight in different functions to ensure proper localization of various enzymes.

*Sorting and processing of MMPs and lysosomal enzymes in the Golgi*—For many years, the importance of other proteases has been overshadowed by the matrix MMPs in the field of cancer metastasis. However, due to the clinical failure of MMP inhibitors to prevent cancer metastasis, additional proteases such as cathepsins have been investigated as potential candidates for cancer metastasis management. Changes to Golgi structure will impact the fidelity of protein sorting intracellularly and the secretion of proteins to the extracellular space (Xiang et al., 2013). While cathepsins are localized primarily in lysosomes, they can be secreted into the extracellular space where they participate in the degradation of ECM proteins. In this manner, cathepsins are also key regulators of basement membrane structure and their catalytic activity outside of the cell can promote tumor cell invasion and metastasis (Gormley et al., 2011) (Yang et al., 2010). Previous studies have demonstrated the key role of different types of cathepsins in cancer cell invasion, such as cathepsin (CTS) B in glioma (Demchik et al., 1999), CTS-L in ovarian carcinoma (Zhang et al., 2014), and CTS-S in colorectal (Burden et al., 2009) and breast cancer metastasis (Sevenich et al., 2014). Cathepsin D elevation is also a poor prognostic marker for breast, ovarian, prostate, bladder, and melanoma cancer, and has been previously associated with increased risk of relapse and metastasis (Dian et al., 2012; Dian et al., 2014). Breast and prostate cell lines can grow and proliferate more quickly in the presence of secreted pro-cathepsin D (Vĕtvicka et al., 1994; Hu et al., 2008). Interestingly, depletion of both GRASP55 and GRASP65 caused missorting of cathepsin D precursors to the extracellular space (Xiang et al., 2013). Upon characterizing these cells lines lacking GRASP proteins, Wang and colleagues observed significant acceleration of cell cycle progression in GRASP55 and GRASP65 knockdown cells when compared to control cells (Tang et al., 2010; Ahat et al., 2019). Altogether, these studies suggest that the secretion of ECM remodeling proteins depends on the Golgi structure that is required for proper sorting, adequate glycosylation, and cleavage activation of the enzymes.

### 3.3 Golgi Adaptation During Metastatic Dissemination

#### 3.3.1 Cancer Cell Survival in the Circulation and Attachment to the Endothelium

In the next step of the metastatic cascade, cancer cells disseminate from the primary tumor to establish new metastases at distant sites mainly through the bloodstream (Figure 2). During hematogenous dissemination, circulating tumor cells encounter normal host cells such as platelets. These interactions can endow tumor cells with the necessary metastatic properties to survive in blood circulation, arrest in vasculature, and establish new tumor growth at the secondary site. In this subsection, we focus on Golgi-localized proteins and their function in cancer cell survival in circulation and attachment to the endothelium.

*Alteration of cell adhesion molecules during metastatic dissemination*—As mentioned earlier, the Golgi-localized protein PAQR11 has been reported to suppress anoikis, a form of programmed cell death induced upon detachment from extracellular matrix to prevent the re-adhesion of the cell in incorrect locations (Tan et al., 2021). By gaining anoikis-resistance, cancer cells survive in circulation and subsequently colonize ectopic sites where ECM proteins differ from the primary site. One of the ways cancer cells can acquire anoikis-resistance is by changing integrins at the cell surface. There are 18 α subunits and 8 β subunits that have been identified in humans, which are able to generate 24 different αβ heterodimeric integrins (Takada et al., 2007). Since integrins function as heterodimers, the numerous combinations mediate binding preference for specific ligands such as fibronectin, laminin, or collagen—thereby permitting malignant cells to grow in distant organs (Walker et al., 2018). Interestingly, disrupting Golgi structure through GRASP55 depletion reduced the protein expression of α5β1 integrin in cervical cancer and breast cancer cells (Ahat et al., 2019). Other integrins such as αvβ3, α1β1 and α6β1, along with α5β1 are all implicated in cell survival (Avraamides et al., 2008).

It is proposed that integrins also protect circulating cancer cells exposed to shearing forces generated by blood flow in the vasculature. Integrin αvβ3 on tumor cells and αIIbβ3 on activated platelets can bind fibrin to form a platelet-rich thrombus around tumor cells which shields the cells from fluid shear stress (Chen et al., 2015). Additionally, the formation of these platelet-rich thrombi may also mask tumor cells antigens from Natural-Killer (NK) cell immune surveillance. When MHC class I from non-cancerous, normal platelets coating the tumor cells is presented, the malignant cell escapes lysis by the innate immune system (Labelle and Hynes, 2012; Schmied et al., 2021). By surviving the initial steps of hematogenous dissemination, those tumor cells have the chance to stably adhere to endothelial cells and extravasate out of the bloodstream to seed the stroma of new sites.

*Golgi alteration and integrin glycosylation during metastatic dissemination*—The Golgi is home to glycosyltransferases and glycosidases that cooperate to support glycosylation of nearly half of the human proteome. Glycan modifications are sequentially carried out by these Golgi enzymes that localized to specific Golgi compartments [cis-, medial-, and trans-Golgi cisternae, and the trans-Golgi network (TGN)] according to their function in the biosynthetic pathway. When the Golgi structure is perturbed through knockdown of GRASP proteins, accurate protein glycosylation was impaired (Zhang and Wang, 2016). GRASP depletion, particularly GRASP55 single depletion or GRASP55/65 double depletion, resulted in a decrease in overall glycan quantity, complexity, and glycoprotein composition at the plasma membrane, based on glycomic analysis by mass spectrometry (Xiang et al., 2013). It has been reported that disrupting Golgi structure in cancers results in an increase of sialyation (Schultz et al., 2012). Increased sialyation of carbohydrate determinants such as Sialyl LewisX (sLe^X^) and sialyl-Lewis a (sLe^a^) on cancer cells promote their binding to selectins which may favor their arrest on endothelia. As expected, high expression of sLe^x^ and sLe^a^ correlated with metastasis and poor survival and by way of contrast, downregulation of these selectin ligands through knockdown of key glycosyltransferases reduced adhesion to selectins and diminished metastatic ability (Schultz et al., 2012). Removal of α2-8 sialic acid residue in the α5 integrin subunit of melanoma cells inhibited cell adhesion to fibronectin (FN), but the opposite was observed for hyposialyation of β1 integrin subunit in myeloid cells which enhanced FN binding. Meanwhile, enrichment of T and sialyl T antigen on β1 integrin enhanced its activity (Liu et al., 2014).

These results demonstrate the differential effects of O-glycosylation on integrins is dependent on the subunit, site, and cell type. In epithelial cells, a shift of N-glycans on integrins to the highly β1, 6 GlcNAc branched type is catalyzed by a resident Golgi enzyme, N-acetylglucosaminyltransferase V (MGAT5), which leads to decreased cell adhesion and promotes cell motility (Murata et al., 2000) (Granovsky et al., 2000). Conversely, an upregulation of N-acetylglucosaminyltransferase III (MGAT3) modifies N-glycans with a bisecting GlcNAc residue, suppresses terminal modifications, and reduces integrin-mediated signaling. Through site-directed mutagenesis studies, it was demonstrated that specific N-glycosylation sites on β3 integrin positively regulates α_IIb_β_3_ but not α_V_β_3_ (Cai et al., 2017). Still, further studies are needed to clarify the structural consequences of individual N-glycan or O-glycan site on integrin activation and to clarify how Golgi unstacking selectively decreases the synthesis of α5β1 integrin.

*Other cell adhesion molecules in metastatic dissemination*—Although integrins represent a class of proteins found on the plasma membrane for cell adhesion to the ECM, it is equally important to highlight cell-cell contact in the metastatic process which can also be regulated via glycosylation. One example is N-glycosylation of EpCAM, a transmembrane glycoprotein primarily used as an epithelial marker and aberrant expression is associated with cancer. EpCAM belongs to a family of cell adhesion molecules (CAMs) that mediates cell-cell contact; however studies have yet to resolve EpCAM’s role in cell adhesion (Gaber et al., 2018). EpCAM expression is generally lower in squamous cell carcinomas than in adenocarcinomas; while absent in most sarcomas, lymphomas, melanomas, and neurogenic tumors (Went et al., 2004). In contrast, the majority of other cancers express EpCAM at levels much higher than normal non-cancerous controls. At the post-transcriptional level, EpCAM expression can be modulated by N-glycosylation since the protein contains three N-glycosylation sites (Asn74, 111, 198), which have been implicated in protein stability (Munz et al., 2008). A later study confirmed the loss of glycosylation at Asn198 reduced protein stability at the membrane (Chong and Speicher, 2001). Glycosylation sites of many other cell surface proteins part of the glycocalyx have yet to be mapped and characterized in detail, despite cancer literature enumerating a vast number of aberrantly glycosylated substrates [reviewed (Fuster and Esko, 2005; Costa et al., 2020)]. As continued discoveries are made towards understanding the cancer glycome, we expand the possibilities of new therapeutic strategies to exploit glycosylation differences on cell surface proteins or glycosylation enzymes themselves found at the Golgi (Bloise et al., 2021) (Daniels et al., 2002). A summary of Golgi enzymes associated with known pro-metastatic alterations in different cancers are provided in Table 1.

**TABLE 1:** Golgi enzymes linked to glycosylation alterations in cancer.

	Gene	Golgi protein	Function	Golgi location	Cancer type	Selected References
	GALNT3	Polypeptide N-acetylgalactosaminyltransferase 3	Catalyzes the initial reaction in O-linked oligosaccharide biosynthesis, the transfer of an N-acetyl-D-galactosamine residue to a serine or threonine residue on the protein receptor	medial/trans	pancreatic cancer	Chugh et al. (2016)
	GALNT2	Polypeptide N-acetylgalactosaminyltransferase 2	Catalyzes the initial reaction in O-linked oligosaccharide biosynthesis, the transfer of an N-acetyl-D-galactosamine residue to a serine or threonine residue on the protein receptor	medial/trans	hepatocellular carcinoma	Wu et al. (2011)
	GALNT6	Polypeptide N-acetylgalactosaminyltransferase 6	Catalyzes the initial reaction in O-linked oligosaccharide biosynthesis, the transfer of an N-acetyl-D-galactosamine residue to a serine or threonine residue on the protein receptor	medial/trans	breast cancer, pancreatic cancer, ovarian endometrioid and clear cell carcinoma	Park et al. (2010); Lin et al. (2017)
	GALNT14	Polypeptide N-acetylgalactosaminyltransferase 14	Catalyzes the initial reaction in O-linked oligosaccharide biosynthesis, the transfer of an N-acetyl-D-galactosamine residue to a serine or threonine residue on the protein receptor	medial/trans	breast cancer, pancreatic cancer, non–small-cell lung cancer, and melanoma	Shan et al. (2018)
	B4GALNT2	Beta-1,4 N-acetylgalactosaminyltransferase 2	Transfers a beta-1,4-linked GalNAc to the galactose residue of an alpha-2,3-sialylated chain	trans	colon cancer, breast cancer	Pucci et al. (2020); Yu et al. (2021a)
	B4GALT3	Beta-1,4-galactosyltransferase 3	Responsible for the synthesis of complex-type N-linked oligosaccharides in many glycoproteins as well as the carbohydrate moieties of glycolipids	trans	neuroblastoma	Chang et al. (2013)
	B4GALT1	Beta-1,4-galactosyltransferase 1	Responsible for the synthesis of complex-type N-linked oligosaccharides in many glycoproteins as well as the carbohydrate moieties of glycolipids	trans	lung cancer, breast cancer, leukemia, colon cancer, hepatocellular cancer, prostate cancer, clear cell renal cell carcinoma,	Wei et al. (2008); Choi et al. (2012); Shinzaki et al. (2012); Zhou et al. (2013); Radhakrishnan et al. (2011); Xie et al. (2016); De Vitis et al. (2019)
	ST6GAL1	Beta-galactoside alpha-2,6-sialyltransferase 1	Transfers sialic acid from CMP-sialic acid to galactose-containing acceptor substrates	trans	stomach cancer, ovarian cancer, colon cancer, colorectal cancer, breast cancer, pancreatic ductal adenocarcinoma, gastric cancer	Kudo et al. (1998); Dall'Olio and Chiricolo (2001); Lise et al. (2000); Sewell et al. (2006); Marcos et al. (2011); Schultz et al. (2013); Chakraborty et al. (2018); Park and Lee (2013); Dalziel et al. (2001)
	ST3GAL2	CMP-N-acetylneuraminate-beta-galactosamide-alpha-2,3-sialyltransferase 2	A beta-galactoside alpha2-3 sialyltransferase primarily involved in terminal sialylation of ganglio and globo series glycolipids	trans	colorectal cancer	Kudo et al. (1998)
	ST3GAL3	CMP-N-acetylneuraminate-beta-1,4-galactoside alpha-2,3-sialyltransferase	Catalyzes the formation of the NeuAc-alpha-2,3-Gal-beta-1,4-GlcNAc-, NeuAc-alpha-2,3-Gal-beta-1,3-GlcNAc- and NeuAc-alpha-2,3-Gal-beta-1,3-GalNAc- sequences found in terminal carbohydrate groups of glycoproteins and glycolipids	trans	T-cell leukemia	Radhakrishnan et al. (2011)
	ST3GAL4	CMP-N-acetylneuraminate-beta-galactosamide-alpha-2,3-sialyltransferase 4	A beta-galactoside alpha2-3 sialyltransferase involved in terminal sialylation of glycoproteins and glycolipids	trans	colorectal cancer	Kudo et al. (1998)
	MGAT5	Alpha-1,6-mannosylglycoprotein 6-beta-N-acetylglucosaminyltransferase A	Catalyzes the addition of N-acetylglucosamine (GlcNAc) in beta 1–6 linkage to the alpha-linked mannose of biantennary N-linked oligosaccharides C-6 of the core α1-6Man	medial/trans	breast cancer, colon cancer	Seberger and Chaney (1999); Granovsky et al. (2000); Guo et al. (2014)
	MGAT3	Beta-1,4-mannosyl-glycoprotein 4-beta-N-acetylglucosaminyltransferase	Catalyzes the addition of N-acetylglucosamine in beta 1–4 linkage to the beta-linked mannose of the trimannosyl core of N-linked sugar chains, called bisecting N-acetylglucosamine (GlcNAc)	medial	lung cancer	Yoshimura et al. (1995)
	MGAT1	Catalyzes the first GlcNAc residue to the C-2 of the α1-3Man in the core of Man5GlcNAc2	Alpha-1,3-mannosyl-glycoprotein 2-beta-N-acetylglucosaminyltransferase	cis/medial	prostate cancer, breast cancer, hepatocellular carcinoma, colorectal cancer	Beheshti Zavareh et al. (2012); Yu et al. (2021b); Takayama et al. (2020); Sethi et al. (2014)
	FUT3	3-galactosyl-N-acetylglucosaminide 4-alpha-L-fucosyltransferase	Catalyzes the transfer of L-fucose, from a guanosine diphosphate-beta-L-fucose, to both the subterminal N-acetyl glucosamine (GlcNAc) of type 1 chain (beta-D-Gal-(1->3)-beta-D-GlcNAc) glycolipids and oligosaccharides via an alpha(1,4) linkage, and the subterminal glucose (Glc) or GlcNAc of type 2 chain (beta-D-Gal-(1->4)-beta-D-GlcNAc) oligosaccharides via an alpha(1,3) linkage, independently of the presence of terminal alpha-L-fucosyl-(1,2) moieties on the terminal galactose of these acceptors	trans	pancreatic cancer	Zhan et al. (2018)
	FUT6	4-galactosyl-N-acetylglucosaminide 3-alpha-L-fucosyltransferase	Catalyzes the transfer of L-fucose, from a guanosine diphosphate-beta-L-fucose, to the N-acetyl glucosamine (GlcNAc) of a distal alpha2,3 sialylated lactosamine unit of a glycoprotein- or a glycolipid-linked sialopolylactosamines chain or of a distal or internal lactosamine unit of a neutral glycoprotein- or a glycolipid-linked polylactosamines chain through an alpha-1,3 glycosidic linkage	trans	breast cancer	Matsuura et al. (1998)
	FUT7	Alpha-(1,3)-fucosyltransferase 7	Catalyzes the transfer of L-fucose, from a guanosine diphosphate-beta-L-fucose, to the N-acetyl glucosamine (GlcNAc) of a distal alpha2,3 sialylated lactosamine unit of a glycoprotein or a glycolipid-linked sialopolylactosamines chain through an alpha-1,3 glycosidic linkage	trans	T cell leukemia	Hiraiwa et al. (2003)
	FUT8	Alpha-(1,6)-fucosyltransferase	Catalyzes the addition of fucose in alpha 1–6 linkage to the first GlcNAc residue, next to the peptide chains in N-glycans	medial	hepatocellular carcinoma, lung cancer, breast cancer	Noda et al. (1998); Wang et al. (2009); Liu et al. (2011); Potapenko et al. (2010)
	C1GALT1C1	C1GALT1-specific chaperone 1	Probable chaperone required for the generation of 1 O-glycan Gal-beta1-3GalNAc-alpha1-Ser/Thr (T antigen), which is a precursor for many extended O-glycans in glycoproteins. Probably acts as a specific molecular chaperone assisting the folding/stability of core 1 beta-3-galactosyltransferase (C1GALT1)	cis	gastric carcinoma, hepatocellular carcinoma	Wang et al. (2010); Ju et al. (2014); Shen et al. (2020)
	C1GALT1	Glycoprotein-N-acetylgalactosamine 3-beta-galactosyltransferase 1	Glycosyltransferase that generates the core 1 O-glycan Gal-beta1-3GalNAc-alpha1-Ser/Thr (T antigen), which is a precursor for many extended O-glycans in glycoproteins	cis	breast cancer	Chou et al. (2015)
	GCNT3	Beta-1,3-galactosyl-O-glycosyl-glycoprotein beta-1,6-N-acetylglucosaminyltransferase 3	Glycosyltransferase that can synthesize all known mucin beta 6 N-acetylglucosaminides. Mediates core 2 and core 4 O-glycan branching	medial	breast cancer, gastric cancer, prostate cancer	Dalziel et al. (2001)
	GCNT2	N-acetyllactosaminide beta-1,6-N-acetylglucosaminyl-transferase	Branching enzyme that converts linear into branched poly-N-acetyllactosaminoglycans	medial	melanoma	Sweeney et al. (2018)

*Alteration of Golgi-mediated lipid synthesis in cancer cells*—Since sphingolipid metabolism contributes to cancer cell survival, efforts have been made towards elucidating direct intracellular protein targets of sphingolipids, with the goal of identifying new therapeutic strategies. Many distinct malignancies have been shown to exhibit dysregulation of sphingolipid metabolism, including bioactive sphingolipids, or SLs, such as ceramide and sphingosine-1-phosphate, which have been involved in cell death, cell survival, and cell proliferation (Hannun and Obeid, 2018).

Alteration of the Golgi structure also affects the synthesis glycolipids. Both globotriaosylceramide (Gb3) and monosialotetrahexosylganglioside (GM1) gangliosides are glycolipids synthesized in the Golgi from the same precursor and then transported to the cell surface. Disruption of the Golgi by knocking out GRASP55 and GRASP65 reduces Gb3 expression but increases GM1 level (Bekier et al., 2017). A more recent study demonstrated that GRASP55 specifically binds and compartmentalizes key glycosphingolipid biosynthetic enzymes; the correct compartmentalization of these enzymes at the Golgi promises accurate biosynthetic reactions and regulates cellular glycosphingolipid profile (Pothukuchi et al., 2020). Gb3 expression is correlated with gastric, colon, and breast cancer progression and Gb3 is associated with mechanisms in the EMT pathway (Cumin et al., 2021). Furthermore, susceptibility to chemoagents have been linked to Gb3. For example, cisplatin-resistant cells showed increased expression of Gb3 at the cell surface (Tyler et al., 2015). Thus, these findings further implicate the role of Golgi in the adapting cancer cell.

#### 3.3.2 Conventional and Unconventional Secretion and Generation of the Pre-metastatic Niche

The distant microenvironment lacking cancer cells, but primed for metastatic colonization due to alterations that condition it to support abnormal tumor-growth is termed the pre-metastatic niche (Peinado et al., 2017). Primary tumor cells create a microenvironment in secondary sites that is conducive to their survival even before they arrive to establish micrometastasis. These pro-tumorigenic cues are brought about by the numerous tumor-secreted factors and tumor-shed extracellular vesicles. These signaling molecules include various growth factors, cytokines, chemokines, and pro-angiogenic factors of the pro-metastatic secretome. Secreted proteins that have N-terminal signal sequences are conventionally transported from the ER to the Golgi and then to the plasma membrane where they are released to the extracellular space. In contrast, some cytosolic proteins lacking an ER signal sequence bypass the Golgi and are secreted through the unconventional secretion pathway (Grieve and Rabouille, 2011). Although the unconventional pathway does not follow the canonical ER-to-Golgi route, GRASP proteins have been implicated by previous studies in unconventional secretion (Kinseth et al., 2007; Nüchel et al., 2021).

*Secretion of pro-metastatic factors*—Many tumor-secreted growth factors activate angiogenesis and/or cell activation and differentiation to prepare the pre-metastatic niche. These include vascular endothelial growth factor A (VEGFA), angiopoietin-like 4 (ANGPTL4) and transforming growth factor-α (TGFα). GRASP55 has been shown to interact with the TGFα (Kuo et al., 2000). TGFα is expressed primarily in ectodermic and epithelial cells during normal development and is frequently up-regulated in carcinoma cells (Greten et al., 2001; Awwad et al., 2003; Calvisi and Thorgeirsson, 2005), and expression of TGFα can confer a transformed phenotype and tumorigenesis to normal cells (Derynck, 1990; Lee et al., 1995; Humphreys and Hennighausen, 2000; Shi et al., 2000). Interference with the interaction between GRASP55 and TGFα strongly impairs cell surface expression of TGFα, suggesting an important role for GRASP55 in TGFα transport and maturation through the Golgi apparatus (Kuo et al., 2000). GRASP55 therefore seems to be a versatile protein that facilitates secretion of growth factors and cytokines supporting the pre-metastatic niche through both the unconventional secretion pathway and conventional secretion pathway.

*GRASP-dependent unconventional secretion*—As described above, some proteins lacking an ER signal sequence can be transported and released by a Golgi-independent but GRASP-dependent unconventional pathway. One such example is interleukin 1 beta (IL-1β) (Zhang et al., 2015). IL-1β has been shown to be upregulated in many solid tumors, including melanoma, colon, lung, breast, or head and neck cancers and is associated with poorer prognosis (Rébé and Ghiringhelli, 2020). IL-1β can modulate gene expression and cytokine production, regulating cellular adhesion and migration, angiogenesis, or immune response. Transforming growth factor-β (TGFβ) and fibroblast growth factor 2 (FGF2) are also released by unconventional secretion. TGFβ-dependent protein synthesis, such as of periostin, plays a key role in the development of the pre-metastatic niche by maintaining future infiltration of metastasis-initiating cells through WNT signaling. Extracellular FGF2 affects proliferation, drug sensitivity, and apoptosis of cancer cells.

### 3.4 Golgi and the Tumor Microenvironment

#### 3.4.1 Golgi Response to Stress

Both primary and secondary tumors are composed of cancer cells and a tumor microenvironment (TME). The tumor microenvironment is comprised of different cell types such as lymphocytes, macrophages, mesenchymal stem cells, adipocytes, endothelial cells, and fibroblasts that are recruited and polarized to a pro-tumor, immunosuppressive phenotype within the tumor microenvironment (Baghban et al., 2020). Non-immune cells also release mitogenic growth factors and pro-angiogenic factors into the TME which further exacerbates the remodeling of the ECM and abnormal vasculature around the tumor. The new vasculature is formed to grant nutrient supply to the tumor; however, as the tumor mass grows, blood supply to the core of the tumor mass is insufficient and the TME becomes hypoxic and nutrient deprived. How does the Golgi respond to cues from the tumor microenvironment?

**FIGURE 4:**
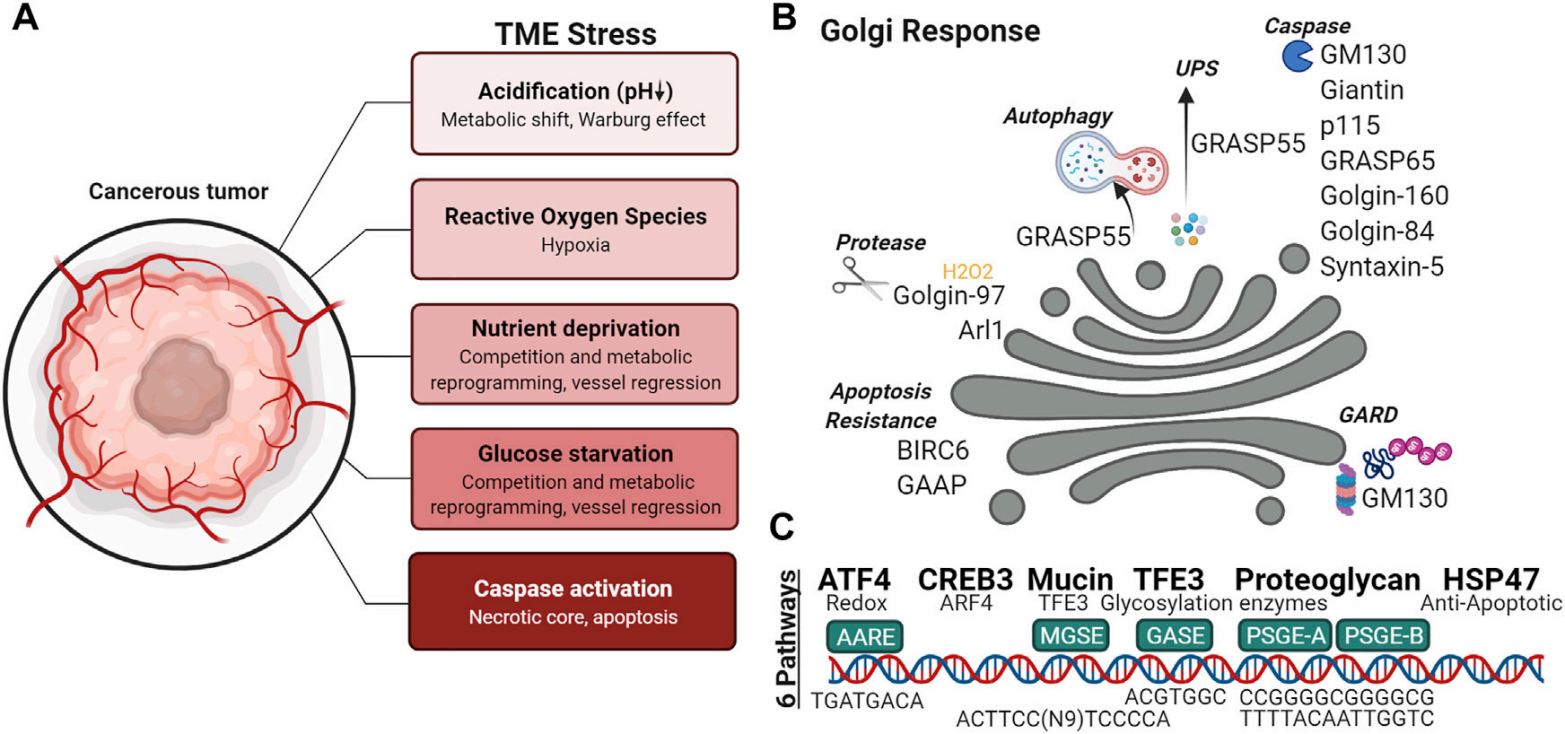
Golgi response to the tumor microenvironment. **(A)** Variety of stresses are found in the tumor microenvironment, **(B)** capable of activating Golgi-associated degradation (GARD), autophagy, unconventional protein secretion (ups), cleavage of Golgi proteins and **(C)** Golgi Stress Response pathways.

*Signal transduction and protein homeostasis at the Golgi in cancer cells*—Many signaling molecules are docked on the Golgi membranes and respond to various cellular stressors, leading to the hypothesis that the Golgi operates as a signal hub (Mayinger, 2011). Nutrient deprivation is a common characteristic of the tumor microenvironment (Figure 4A); under such stresses, autophagy is a catabolic mechanism that helps cancer cells adapt to metabolic needs during nutrient deprivation, genotoxic stress, growth factor withdrawal and hypoxia. Under glucose starvation and amino acid starvation conditions, a subpopulation of GRASP55 is retargeted from the Golgi to the interface between autophagosomes and lysosomes (Figure 4B) to promote autophagosome maturation (Zhang et al., 2018). Interestingly, GRASP55 O-GlcNAcylation is reduced under glucose deprivation causing the protein to relocalize to autophagosome-lysosome membranes, demonstrating a link between physiological stress signaling and nutrient sensing by the highly adaptive organelle. Cellular stress such as nutrient deprivation can also activate pro-survival autophagy, which is linked to the loss of Golgi integrity (Núñez-Olvera et al., 2020). Golgi stress, like ER stress, can cause proteasomal degradation, in attempts to restore Golgi proteostasis. Similar to ERAD (ER-associated degradation), if Golgi stress persists, Golgi-apparatus related degradation (GARD) may be triggered (Figure 4B). GM130 is a degraded locally at the Golgi by cytosolic proteasomes in the presence of Golgi stress, resulting in Golgi dispersal (Eisenberg-Lerner et al., 2020).

*Alteration of Golgi pH and glycosylation under stress*—Due to shortages in nutrient in the TME, cancer cells undergo a metabolic switch, and favor the fermentation of glucose to lactate, and thus, lowering the pH of the microenvironment (Figure 4A). A normal, healthy Golgi maintains a strict pH gradient across the Golgi stack, where the *cis*-Golgi has a pH of 6.7 and progressively becomes more acidic at *trans-*Golgi with a pH of 6.0 (Kellokumpu, 2019). To maintain the distinct pH set points, the Golgi employs ion channels to maintain its homeostasis. Differential expression of ion channels is commonly observed in cancer. Recently NHE7, an NHE family ion channel responsible for the maintenance of cytosolic pH, was shown to regulate lumenal acidification of the TGN. Intriguingly, NHE7 deficiency disrupted protein sialylation in pancreatic ductal adenocarcinoma (Galenkamp et al., 2020). Even moderate Golgi pH alterations such as those detected in cancer cells can impair N-glycosylation by inducing selective mislocalization of certain Golgi glycosyltransferases. Of the Golgi glycosyltransferases tested by Kellokumpu and colleagues, alpha(2,3)-sialyltransferase (ST3) was more sensitive to pH changes than alpha(2,6)-sialylation (ST6), beta(1,4)-galactosylation (B4GalT1) or alpha-Mannosidase II (ManII) (Kellokumpu et al., 2002). When the Golgi enzymes are localized to inappropriate compartments, they are less likely to encounter their substrates. Furthermore, since some glycosylation enzymes are known to increase their catalytic function when forming heterodimers, spatially separation of the enzymes into different Golgi and post-Golgi compartments will hinder their oligomerization and functional efficiency. For example, dimerization of ST6 and B4GalT1 permits their full catalytic activity, whereas the absence of this interaction is associated with cancers and hypoxia (Khoder-Agha et al., 2019).

*Golgi adaptation to oxidative stress*—Within the microenvironment (Figure 4A), oxidative stress may have intrinsic or extrinsic origins. Some stromal components can directly produce reactive oxygen species (ROS), while hypoxia produces oxidant species via dysregulation of the mitochondrial electron transport or of NADPH oxidase activity. A recent study using hydrogen peroxide treatment to study ROS-induced oxidative stress, revealed *trans-*Golgi membrane tethers are targets of ROS. Golgi degradation of Arl1 and dissociation of GRIP domain-containing proteins Golgin-97 and Golgin-245 from the trans-Golgi was observed when cells were treated with hydrogen peroxide (Ireland S. et al., 2020) (Figure 4B). Hypoxia has also been shown to down- or up-regulate enzymes that synthetize nucleotide sugars in the cytoplasm, Golgi-localized glycosyltransferases, fucosyltransferases, and sialyltransferases (Kellokumpu, 2019). Additionally, activation of the hypoxia inducible factor-1α (HIF-1α)/heme-oxygenase-1 (HO-1) pathway by oxidative stress upregulates the expression of Golgi proteins: GM130, Golgin-97 and mannosidase II (Li et al., 2021). It has been proposed that upregulation of Golgi associated genes during cellular stress serves to increase Golgi function as a compensatory mechanism.

*Transcriptional regulation during Golgi stress response*—How the Golgi responds to stress and integrates stress signals remains unclear (Figure 4C). While it is known that activation of CREB3 and CREB3L, transcription factors of the Golgi-associated genes ARF4, COPB1, GGA2, and USO1, is increased in metastatic cells (Howley et al., 2018) (Reiling et al., 2013), other Golgi stress response pathways and regulators of these pathways have yet to be described in full mechanistic detail. Currently, six response pathways of the Golgi stress response have been reported in mammalian cells: TFE3, PGSE proteoglycan, MAPK-ETS, mucin, CREB3, and HSP47 pathways (Sasaki and Yoshida, 2019). TFE3 binds a Golgi apparatus stress response element (GASE) in the promoter of Golgi-associated genes, to activate the transcription of some glycosyltransferases (Taniguchi et al., 2015), Golgi structural proteins GCP60, Giantin, and GM130, proteins involved in vesicular transport such as Syntaxin 3A (STX3A), RAB20 (Ras-related protein Rab-20), and Golgi proteases (Oku et al., 2011; Sasaki and Yoshida, 2019). Proteoglycan-type Golgi stress response element (PGSE) is present on the promoters of glycosyltransferases (e.g., B3GAT3, EXT2 and CSGALNACT2), an isomerase (GLCE) and sulfotransferases (HS6ST1, HS3ST1, NDST2 and CHST7) (Sasaki et al., 2019). Sensors for each Golgi stress pathway has not been elucidated. The concept of the Golgi stress response is still an active area of research with many outstanding questions. Further understanding of the Golgi stress response (Figure 4) will undoubtedly lead to a more complete view of the role of Golgi in cancer metastasis.

#### 3.4.2 Apoptosis and Golgi

When cellular stress exceeds the compensatory limits of the cell, the apoptotic cascade is ushered in by the activation of caspases. During apoptosis, caspase-mediated cleavage of GRASP65 results in fragmentation of the Golgi (Lane et al., 2002). Specifically, cleavage of GRASP65 (Figure 4C)was shown to promote Fas/CD95-mediated apoptosis via release of C-terminal fragments that act at the mitochondria (Cheng et al., 2010). Other golgins such as Golgin-160 are cleaved by Golgi-localized caspase-2 during apoptosis (Mancini et al., 2000; Maag et al., 2005). GM130, GCC88, p115, Golgin-84, Syntaxin 5, and Giantin are also cleaved into distinct fragments (Figure 4C); some capable of acting as signaling molecules, similar to the aforementioned cleavage fragment of GRASP65 (Lowe et al., 2004). For example, p115 caspase cleavage fragment (Figure 4C) promotes apoptosis through the ERK/p53/PUMA pathway (How and Shields, 2011). During malignant transformation, anti-apoptotic kinases are increased and targeted to the Golgi; often coinciding with Golgi dispersal that is thought to support tumor growth by preventing the arrival of pro-apoptotic signaling kinases to the Golgi (Sundram et al., 2011). The apoptotic activity of PKC-theta regulatory domain is dependent on Golgi localization (Schultz et al., 2003). Conversely, anti-apoptotic proteins can be found at the Golgi, such as BIRC6 and GAAP (Golgi anti-apoptotic protein). BIRC6 is found at the *trans*-Golgi where it binds activated caspase 9/SMAC via its BIR motif and through its UBC domain ubiquitinates the two apoptotic proteins for degradation (Hao et al., 2004). Increasing evidence shows that Golgi is involved in apoptotic signaling. Therefore, mechanisms that promote or inhibit these signals in the Golgi may be worthwhile for researchers to look into given that many anti-cancer treatments result in classical caspase-dependent apoptosis.

## 4 Future Direction and Concluding Remarks

Over 50 years ago, ultrastructural images of the Golgi in cancer cells revealed the hypertrophic morphology of this fascinating, dynamic organelle (Maldonado et al., 1966). Throughout the years, the concept of the Golgi has evolved from being an intermediary station where proteins are trafficked before reaching their final destinations, to being recognized as a signaling hub and responsive component of the endomembrane system that is integral to many cellular functions – such as cell proliferation, cell migration, proteostasis, and apoptosis. Primed to both receive cues from the extracellular environment and to act as the orchestrator of cellular events, the definition of a cancer cell Golgi therefore remains to be refined.

We have only begun to scratch the surface of how the Golgi contributes to cancer metastasis. Golgi function is intimately connected to Golgi structure. Interestingly, both Golgi compaction and Golgi fragmentation have been reported in cancer cells. While most cancer cell lines in culture have an intact Golgi ribbon, future research should investigate the Golgi structure in 3D models that better represent tumor microenvironment and cancer cell progression. Our speculation that Golgi alteration facilitates cancer biogenesis is based on the following reasons: 1) The Golgi undergoes morphological change in tissue culture upon the induction of EMT (Tan et al., 2017) or migration (Bisel et al., 2008b); 2) Golgi fragmentation is often seen in cancer tissues (Maldonado et al., 1966; Tani et al., 1975; Kellokumpu et al., 2002; Sano et al., 2002; Petrosyan, 2015; Zhang, 2021); 3) Alteration of Golgi functions such as glycosylation is a hallmark of cancer cells (Zhang, 2021). 4) While an organized Golgi structure is required for accurate protein glycosylation and sorting, our study revealed that Golgi fragmentation accelerates protein trafficking and cell proliferation (Xiang et al., 2013; Zhang and Wang, 2016; Bekier et al., 2017). Therefore, although the current literature has not provided solid evidence that Golgi morphological changes contribute to cancer initiation, it is possible that they may facilitate cancer progression.

As new findings emerge, it will be important to address the heterogeneity of cancer phenotypes. Is this heterogeneity due to different stressors eliciting different Golgi responses? How does the Golgi sense these chemical and mechanical changes in the tumor milieu? Do the Golgi architectures vary at different stages of cancer progression, across different cell types, and perhaps even within different regions of the same tumor (e.g., leading edge, necrotic core)? More systematic studies of patient tissues, patient-derived primary cell organoids, 3D culture models, and co-culture systems will help answer these questions. Research on Golgi adaptations to cancer-associated stresses and signaling cues holds great promise for a more conclusive understanding of the link between Golgi and cancer. In addition, with many screening methods now available, such as CRISPR, proximity ligation and inducible degradation, new Golgi functions will be discovered. Furthermore, with innovative approaches such as metabolic glycoengineering (MGE), future studies leveraging the alterations of cancer cellular metabolism to modulate glycosylation at the cell surface, might be useful for studying cancer cells with Golgi glycosyltransferase defects. There is abundant evidence to support Golgi structure alteration and glycosylation defects in cancer, although a solid cause-and-effect relationship between these two factors has not been established yet.

Understanding Golgi structure and function through identifying additional roles of Golgi proteins localized to other compartments of the cell, will pave the way for new avenues to study the relevance of Golgi in cancer metastasis and potentially lead to clinical applications for improving patient outcomes. In sum, the Golgi is an important organelle in cancer biology, and we anticipate Golgi dynamics in malignancies will remain an attractive target for future studies.
